# Self-compassion: no communal alternative to agentic self-esteem

**DOI:** 10.3389/fpsyg.2025.1645815

**Published:** 2025-09-18

**Authors:** Nicole Hauke-Forman, Marlene Kollmayer

**Affiliations:** ^1^Institute for Psychology, Friedrich-Alexander-Universität Erlangen-Nürnberg, Erlangen, Germany; ^2^Institute for Psychology of Development and Education, University of Vienna, Vienna, Austria

**Keywords:** agency, communion, self-concept, self-compassion, self-esteem

## Abstract

Research on associations of the two fundamental dimensions of self-concept and positive attitudes toward one’s self has primarily focused on self-esteem which has been shown to be dominantly explained by agency and not communion. The present research included self-compassion as an alternative positive self-attitude construct, characterized by self-kindness rather than positive self-evaluations. Based on theoretical considerations and previous results, we expected self-compassion to be equally predicted by communion and by agency. Two studies came to very similar results: Self-compassion was strongly related to agency, especially to its facet assertiveness. In contrast, self-compassion was only weakly related to communion. On the level of self-compassion’s subcomponents, only self-kindness was related to communion, especially to its facet warmth. Self-esteem was most strongly predicted by agency, especially by its facet assertiveness; competence and warmth showed additional weaker predictive power. Thus, self-compassion seems to be an alternative, but no communal alternative to self-esteem.

## Self-compassion: a communal alternative to agentic self-esteem?

A positive attitude toward the self is important for psychological functioning. Research in this domain has primarily focused on *self-esteem* as a measure of psychological health. High self-esteem, for example, is related to happiness (Cheng and Furnham, 2004), higher positive and lower negative affect (Orth et al., 2012), lower rates of depression (Orth et al., 2009) as well as academic self-concept and academic achievement (Trautwein et al., 2006). But what is our self-esteem, i.e., our evaluative self-perception, based on? Partially on our self-concept, i.e., our descriptive self-perception on the level of traits. The self-concept is often described using the two fundamental dimensions of social cognition, *agency* and *communion* (Abele and Wojciszke, 2014). Agency focusses on the own person and the achievement of personal goals. Typical agentic traits are *intelligent, competent*, and *assertive*. Communion focusses on the integration of an individual into a larger social entity and on social interactions with typical traits being *friendly, caring*, and *honest*. Research has shown that general self-esteem is dominantly explained by agency (e.g., Abele et al., 2016; Wojciszke et al., 2011), while only special components of self-esteem – like relational self-esteem – are related to communion (Hauke and Abele, 2020). However, communal aspects are usually higher and more pronounced than agentic aspects of self-concepts (for an overview see Hauke and Abele, 2019). Why is this the case? Are we only communal because other people value our communal qualities (Abele and Wojciszke, 2014; Hauke and Abele, 2019), or is our communion also of major importance for feeling good about ourselves which is not depicted in self-esteem measures? To answer this question, the present research focuses on an alternative construct implying a positive, healthy attitude toward oneself: *self-compassion*. Self-compassion entails treating oneself with kindness and understanding, perceiving one’s experiences as part of the larger human experience, and being mindful of negative experiences (Neff, 2003b). In the present study, we investigate self-compassion as a possible communal alternative to agentic self-esteem. Therefore, we will examine if self-compassion is a positive attitude toward ourselves that is strongly connected to our communal self-concept.

## The agency-communion-framework

The content dimensions of agency and communion (the “fundamental dimensions” or “Big Two”) are basic to many psychological phenomena, among others, to the self-concept (for a review, see Abele and Wojciszke, 2014). Agency focuses on the individual person and the pursuit of personal goals. Themes of agency are self-realization, striving for power and status, and acting in one’s own interest (Bakan, 1966). Therefore, it is also called the dimension for “getting ahead” in life (Hogan, 1982). Communion focuses on community and social integration. Themes of communion are the formation and maintenance of social relationships, striving for harmony, and acting in the interest of others. Thus, it is the dimension for “getting along” (Hogan, 1982). Agency and communion reflect the two recurring challenges of human life: pursuing individual goals and belonging to social groups (Ybarra et al., 2008).

These twofold conceptualization of content can also be found in many other areas of psychology (for a review, see Abele and Wojciszke, 2014). For example, there are the gender stereotypes of *masculinity* and *femininity* (Bem, 1974; Spence et al., 1974). Masculinity resembles the agency dimension, while femininity resembles the communion dimension. Another example is the cultural difference in *self-construal* (Markus and Kitayama, 1991). Many people in Western cultures construe themselves *independently*, i.e., they emphasize their separateness from others, their internal attributes, and their uniqueness. In contrast, many people in Asia or Latin America construe themselves *interdependently*, i.e., they stress their connectedness to others, their social context, and their relationships. Independence resembles the agency dimension, while interdependence resembles the communion dimension.

Recent research has moved ahead by distinguishing two facets within each “big dimension” (Abele et al., 2016). Agency comprises the facets *agency-competence* (AC) and *agency-assertiveness* (AA), as successful goal pursuit or “getting ahead” requires both skill (competence) and motivation/volition (assertiveness). Communion comprises the facets *communion-warmth* (CW) and *communion-morality* (CM), as establishing and maintaining social relationships or “getting along” requires friendly (warmth) and trustworthy (morality) behavior.

## Self-esteem

Self-esteem, defined as the feeling of self-worth (Rosenberg, 1965), is a fundamental appraisal of the self and relates to the overall value a person places on the self. Research analyzing the relationship between self-concept and self-esteem has shown multiple times that self-esteem is dominated by agency (for a review, see Abele and Hauke, 2018). Regarding the facets, self-esteem is most strongly related to AA (Abele et al., 2016).

Previous research analyzing the relationship between agency, communion, and self-esteem has mainly focused on global self-esteem but the global evaluation of a person can be further differentiated. Within the *Tripartite Model*, three different dimensions of self-esteem are distinguished (Breckler and Greenwald, 1986): personal, relational, and collective self-esteem. *Personal self-esteem* is based on one’s individual attributes like competence and talents and is often measured with the classical Rosenberg scale (1965). *Relational self-esteem* is based on relationships with significant others like family and best friends. *Collective self-esteem* is based on the value of one’s social group like nationality and ethnicity. In contrast to the results regarding personal self-esteem, relational self-esteem shows a strong association with communion in addition to its association with agency. Concerning the facets, AA, CW, and CM predict relational self-esteem (Hauke and Abele, 2020). Soral and Kofta (2020) found collective self-esteem to be better predicted by CM than by AC and CW (however, this single research project testing the relationship between agency, communion, and collective self-esteem did not incorporate AA which seems to be the dominating facet for self-esteem).

Taken together, agency is important for most aspects of self-esteem and dominates personal self-esteem, while communion only plays a role for special components of self-esteem like relational and collective self-esteem. Although all three selves are important and meaningful and are associated with psychological and physical health benefits, research has revealed that people personally value their personal self more than their relational self and their collective self (for a review see Sedikides et al., 2013). Thus, communion does not seem to play an important role for a positive attitude toward the own self if we only consider the research on self-esteem. But are we thinking too restricted by our narrow concentration on self-esteem? Is there an alternative conceptualization of a positive and healthy attitude toward ourselves?

## Self-compassion

Self-compassion is a relatively young psychological concept but has a long history. While its origins lie in Buddhist philosophy going back centuries, self-compassion as a psychological construct was described by Kristin Neff in the early 2000s (Neff, 2003b) who also developed the associated Self-Compassion Scale (SCS, Neff, 2003a). From the beginning, self-compassion was conceptualized as an alternative to self-esteem by defining a different way of a healthy attitude toward oneself (Neff, 2011). While a person’s self-esteem stems from evaluating personal performances in comparison to others or to set standards, self-compassion does not involve such evaluations. In contrast, self-compassion builds on the idea that we should give ourselves the same kindness and care we give to good friends.

The concept of self-compassion consists of three basic components that are related to the way individuals react to personal failure or suffering – self-kindness, common humanity, and mindfulness (Neff, 2003a, 2003b). Self-kindness is defined as treating oneself kind and understanding instead of harsh and judgmental, common humanity means viewing one’s experiences of failure as part of the human condition rather than feeling separated and isolated by them, and mindfulness implies being aware of painful thoughts and feelings instead of over-identifying with them and feeling overwhelmed. These components of self-compassion are distinct on a conceptual and phenomenological level, but they are also interrelated and enhance each other (Neff, 2003b). Meta-analyses found self-compassion to have positive associations to cognitive, psychological, and affective well-being (Zessin et al., 2015), emotional well-being (Bluth and Blanton, 2015), physical health and health behavior (Phillips and Hine, 2021), and self-efficacy (Liao et al., 2021), as well as negative associations to psychopathology (MacBeth and Gumley, 2012) and suicidal thoughts and behaviors and non-suicidal self-injury (Suh and Jeong, 2021). Moreover, self-compassion has a protecting moderating effect on the relationship between neuroticism and depression (Wang and Wu, 2024).

## Similarities and differences between self-compassion and self-esteem

As already described, self-compassion is similar to self-esteem by representing a positive attitude toward the own self and both constructs have lots of positive psychological consequences for an individual’s self and identity. Barnes and Mongrain (2020) found the two constructs to be significantly correlated (*r* = 0.45). Moreover, mindfulness which is an important facet of self-compassion has been found to positively predict self-esteem (Pepping et al., 2013). However, there are also crucial aspects in which the two constructs differ (Neff and Vonk, 2009). Self-esteem often depends on the successful attainment of goals or on appearing superior in social comparisons. Therefore, individuals motivated to maintain high self-esteem often engage in behaviors to defend their egos (for a review see Crocker and Park, 2004). That is why they reject true negative feedback and attribute own failures to external causes. They can also become angry and aggressive toward those who threaten their ego (Baumeister et al., 1996). Self-compassion, in contrast, is a type of open-hearted awareness that can embrace all aspects of personal experience, including failures. This renders ego-defense unnecessary. Moreover, self-esteem is often defined by the feeling of being special and of standing out in the crowd. Conversely, self-compassion entails the feeling of shared human experiences and of being similar to others.

Another problematic feature of self-esteem is that while there are numerous interventions to boost self-esteem, their relative efficacy and the characteristics that moderate this efficacy remain unclear. In their meta-analysis of 119 studies, Niveau et al. (2021) found only small effects of interventions on adults’ global self-esteem, regardless of their format, the target population, and the length of the intervention. In contrast, a recent meta-analysis found that self-compassion interventions for adults and adolescents have a moderate effect on improving self-compassion across 27 studies in different cultural contexts (Ferrari et al., 2019). Research assessing both constructs has shown that self-compassion predicts unique variance in depression and anxiety when controlling for self-esteem (Neff, 2003a). In contrast to self-esteem, self-compassion is not associated with narcissism (Neff, 2003a; Neff and Vonk, 2009). Self-compassion predicts more stable feelings of self-worth than self-esteem and is less contingent on particular life outcomes (Neff and Vonk, 2009). While self-esteem is related to well-being due to feelings of superiority and self-confidence, self-compassion’s relations to well-being stem from feeling safe and secure. In general, self-compassion seems to entail many of the psychological benefits associated with self-esteem but does not involve the tendencies toward self-centeredness critics associated with high self-esteem (Crocker and Park, 2004; McMillan et al., 1994). These results suggest that self-compassion may be a useful alternative to self-esteem for defining what makes up a positive and healthy attitude toward the own self, and that self-compassion might be more related to communal aspects of the self-concept than self-esteem.

## Present research and hypotheses

In the present research, we want to empirically test if self-compassion is a *communal* alternative to the agentic concept of self-esteem. Therefore, we examined the association between the agentic and communal self-concept with self-compassion (Studies 1 and 2) and also with self-esteem (Study 2). Derived from theory, we had the initial hypotheses that self-esteem is strongly related to agency, whereas self-compassion is strongly related to communion. This idea was also already expressed by Neff and Vonk (2009, p. 28). Neff (2006) could already show that self-compassion is predictive of positive relationship behavior and attachment security. The prediction regarding self-compassion also becomes evident from the statement by Neff and McGehee (2010, p.226) that self-compassion is “compassion turned inward” combined with the fact that compassion and kindness are communal constructs.

According to our literature search, we are not aware of any studies that directly tested the association between agency, communion, and self-compassion. However, we found a few studies analyzing the association between self-compassion and constructs akin to agency and communion. In the USA, i.e., an individualistic country (like Germany, where we collected our data), self-compassion was predicted by an independent self-construal (akin to agency), but not by an interdependent self-construal (akin to communion; Neff et al., 2008). Regarding gender role self-concept, both femininity (akin to communion) and masculinity (akin to agency) were found to be significantly related to self-compassion, but masculinity was a stronger predictor for self-compassion than femininity (Patzak et al., 2017; Tatum, 2012; Yarnell et al., 2019). Considering these empirical results showing a strong association between agentic constructs and self-compassion, we revised our theoretically derived hypothesis for self-compassion accordingly: We expected self-compassion to be predicted by communion (based on theoretical considerations) as well as by agency (based on previous empirical results).

Our studies contribute to former research by presenting the first data on the relationship between agency, communion, and self-compassion. By utilizing the constructs of agency and communion and measuring them with the Agency-Communion-Inventory (AC-IN; Abele et al., 2016), we used a more contemporary conceptualization of self-concept and a more modern measurement scale than the previous studies using gender roles measured via the Bem Sex Role Inventory (BSRI; Bem, 1974) and the Personal Attributes Questionnaire (PAQ; Spence et al., 1974; for criticism on these measures see also Hoffman and Borders, 2001; Yarnell et al., 2019). Moreover, the use of the AC-IN also allowed us to test the association between self-compassion and the different facets of agency and communion, i.e., *agency-competence* and *agency-assertiveness,* as well as *communion-warmth* and *communion-morality*. Another strength of the present studies is that we also analyzed the association with self-compassion on the level of its six subscales. In addition, we assessed personal and relational self-esteem in Study 2, so that we could compare the associations between self-concept and self-compassion and between self-concept and self-esteem within one sample to rule out sample-specific effects when simply comparing our results regarding the associations with self-compassion to former research analyzing the associations with self-esteem using different samples.^1^

### Transparency and openness

We report how we determined our sample sizes, all data exclusions (if any), and all measures in all studies. All power and sensitivity analyses were conducted with G*Power (Faul et al., 2007). The data and materials are available at https://osf.io/srjf3/?view_only=db89af10ddcb45859d71b14bb514d9a9.

## Study 1

In Study 1, we analyzed the relationship between agency, communion, and self-compassion. We expected that self-compassion will be predicted by communion as well as by agency. We analyzed the association on the level of agentic and communal facets and of self-compassion subscales in an explorative way.

### Method

#### Sample

To detect a medium effect size (*f^2^* = 0.15) in linear multiple regression (fixed model, single regression coefficient) with four predictors (i.e., two agentic and two communal facets), *α* = 0.05, and a power of 0.95, the minimum sample size needed is 89. Since the magnitude of a correlation can be expected to be stable at a sample size approaching 250 participants (Schönbrodt and Perugini, 2013), we aimed for a sample of approximately this size. We distributed the invitation to the online study via emails and messaging apps using a snowball system, resulting in 263 participants. One participant withdrew his data at the end of the study, leaving us with a final sample of 179 women and 83 men (*M*_age_ = 36.20 years, *SD* = 15.48, range: 18–76). Most participants were employed (48.1%) or students (35.9%). Participants were rewarded with course credit or could take part in a lottery of five vouchers of 10€ each for a wellness store. In a sample of this size, an effect size of *f*^2^ = 0.05 (small to medium) can be detected.

#### Procedure

The online study took about 10 min per participant. After informed consent, participants first rated their agency and communion and then their self-compassion. Finally, we assessed sociodemographic information.

#### Measures

Agentic and Communal Traits. Agency (*α* = 0.84) and communion (*α* = 0.81) together with their two facets each were measured with the AC-IN (Abele et al., 2016). Participants were asked to indicate how much the respective traits apply to them. Each subscale comprised 5 items (sample items: “friendly” [CW; *α* = 0.82], “trustworthy” [CM; *α* = 0.62], “have leadership qualities” [AA; *α* = 0.75], “intelligent” [AC; *α* = 0.80]). The items were presented in a bipolar format with a 7-point Likert-scale (e.g., very unfriendly --- 3-2-1-0-1-2-3 --- very friendly). The bipolar scales were later recoded to 1 to 7 with higher ratings representing the positive pole of the trait (i.e., “very friendly” in the example). Traits were presented in mixed order.

Self-Compassion. Self-compassion (*α* = 0.90) was measured with the 26 items of the Self-Compassion Scale of Neff (2003a) in the German version of Hupfeld and Ruffieux (2011). Participants were asked to indicate how often they acted in the manner stated in each of the items. The scale consists of six subscales (sample items in brackets): self-kindness (“I’m kind to myself when I’m experiencing suffering.,” 5 items; *α* = 0.78), self-judgment (“When I see aspects of myself that I do not like, I get down on myself.,” 5 items; *α* = 0.78), common humanity (“I try to see my failings as part of human condition.,” 4 items; *α* = 0.67), isolation (“When I fail at something that’s important to me I tend to feel alone in my failure.,” 4 items; *α* = 0.78), mindfulness (“When something upsets me I try to keep my emotions in balance.,” 4 items; *α* = 0.67), and over-identification (“When something upsets me I get carried away with my feelings.,” 4 items; *α* = 0.69). The items were answered on a 5-point Likert-scale from 1 = *almost never* to 5 = *almost always*. For calculating the overall self-compassion score, the items of the negative subscales (self-judgment, isolation, and over-identification) were reverse coded. The items were presented in mixed order.

### Results

#### Preliminary analyses

Table 1 shows the descriptive statistics of and Table 2 the correlations between the measured variables.

**Table 1:** Means and standard deviations of the measured variables.

Variables	Study 1 (*N* = 262)		Study 2 (*N* = 434)	
	*M*	*SD*	*M*	*SD*
Agency	5.23	0.78	5.15	0.86
Agency-Assertiveness	4.94	0.97	4.78	1.07
Agency-Competence	5.51	0.77	5.52	0.83
Communion	5.98	0.64	5.96	0.67
Communion-Morality	6.10	0.61	6.08	0.66
Communion-Warmth	5.87	0.84	5.85	0.84
Self-Compassion	3.21	0.56	3.07	0.60
Self-Kindness	3.15	0.73	3.04	0.79
Self-Judgment	2.84	0.77	3.00	0.80
Common Humanity	3.05	0.78	2.93	0.80
Isolation	2.49	0.90	2.69	0.91
Mindfulness	3.28	0.68	3.17	0.73
Over-Identification	2.89	0.82	3.02	0.76
Personal Self-Esteem			3.80	0.81
Relational Self-Esteem			4.00	0.71

**Table 2:** Correlations between the measured variables (Study 1 above and Study 2 below the diagonal).

Variables	1	2	3	4	5	6	7	8	9	10	11	12	13	14
1. Agency	–	0.92***	0.87***	0.12	0.19**	0.04	−0.41***	−0.30***	−0.30***	−0.18***	−0.33***	−0.32***	−0.32***	
2. Agency-Assertiveness	0.93***	–	0.60***	0.07	0.14*	−0.01	−0.44***	−0.29***	−0.33***	−0.18***	−0.40***	−0.31***	−0.38***	
3. Agency-Competence	0.88***	0.65***	–	0.15*	0.21***	0.08	−0.26***	−0.23***	−0.19***	−0.13***	−0.17***	−0.27***	−0.16***	
4. Communion	0.36***	0.26***	0.40***	–	0.81***	0.91***	−0.11***	−0.18***	−0.06***	−0.12***	−0.04***	−0.10***	−0.01***	
5. Communion-Morality	0.38***	0.29***	0.43***	0.86***	–	0.48***	0.13***	0.13***	−0.09***	0.10***	−0.09***	0.13***	−0.03***	
6. Communion-Warmth	0.27***	0.20***	0.31***	0.92***	0.59***	–	−0.07***	−0.18***	−0.02***	−0.11***	−0.01***	−0.06***	−0.04***	
7. Self-Compassion	0.49***	0.48***	0.40***	0.25***	0.25***	0.21***	–	0.76***	−0.80***	0.52***	−0.78***	0.71***	−0.73***	
8. Self-Kindness	0.37***	0.34***	0.33***	0.26***	0.21***	0.25***	0.81***	–	−0.55***	0.47***	−0.41***	0.52***	−0.30***	
9. Self-Judgment	−0.38***	−0.39***	−0.29***	−0.21***	−0.20***	−0.18***	−0.78***	−0.60***	–	−0.20**	0.64***	−0.36***	0.57***	
10. Common Humanity	0.29***	0.28***	0.24***	0.18***	0.15**	0.16***	0.67***	0.59***	−0.35***	–	−0.17**	0.37***	−0.13*	
11. Isolation	−0.41***	−0.40***	−0.35***	−0.22***	−0.22***	−0.18***	−0.78***	−0.45***	0.60***	−0.33***	–	−0.46***	0.65***	
12. Mindfulness	0.38***	0.37***	0.32***	0.16***	0.16***	0.13**	0.72***	0.60***	−0.36***	0.45***	−0.43***	–	−0.49***	
13. Over-Identification	−0.37***	−0.39***	−0.26***	−0.11*	−0.17***	−0.04	−0.75***	−0.42***	0.57***	−0.32***	0.62***	−0.48***	–	
14. Personal Self-Esteem	0.70***	0.66***	0.60***	0.36***	0.35***	0.31***	0.76***	0.59***	−0.68***	0.41***	−0.67***	0.50***	−0.55***	–
15. Relational Self-Esteem	0.50***	0.45***	0.46***	0.37***	0.32***	0.33***	0.41***	0.36***	−0.31***	0.30***	−0.38***	0.27***	−0.23***	0.59***

*p < 0.05; **p < 0.01; ***p < 0.001.

#### Hypothesis testing

We calculated linear regressions with (a) agency and communion and (b) their four facets as predictors for self-compassion and its six subscales. The results of these 14 regressions are shown in Table 3. As expected, agency significantly predicted self-compassion. However, in contrast to our hypothesis, communion was no significant predictor for self-compassion.

**Table 3:** Self-compassion regressed on agency/communion and the four facets (study 1).

Predictors	Self-Compassion	Self-Kindness	Self-Judgment	Common Humanity	Isolation	Mindfulness	Over-Identification
Agency/communion							
*F*	26.21***	16.00***	12.91***	5.63**	16.28***	15.81***	14.95***
*R*^2^ corr.	0.16	0.10	0.08	0.03	0.11	0.10	0.10
A	0.40***	0.28***	−0.30***	0.17**	−0.33**	0.32***	−0.32***
C	0.07	0.15*	−0.02	0.10	−0.01	0.06	0.05
Facets							
*F*	16.22***	8.79***	8.03***	3.09*	13.52***	8.00***	11.79***
*R*^2^ corr.	0.20	0.11	0.10	0.03	0.16	0.10	0.16
AA	0.44***	0.25**	−0.33***	0.17*	−0.47***	0.23**	−0.44***
AC	−0.02	0.07	0.02	0.02	0.12	0.12	0.01
CM	0.05	−0.01	−0.05	0.03	−0.06	0.07	−0.01
CW	0.05	0.18**	0.01	0.09	0.02	0.02	0.03

Displayed are the standardized regression coefficients of linear regressions.

A = agency, C = communion, AA = agency-assertiveness, AC = agency-competence, CM = communion-morality, CW = communion-warmth.

*p < 0.05; **p < 0.01; ***p < 0.001.

#### Explorative analyses regarding the facets and the subscales

Regarding the associations between the two basic dimensions and self-compassion on the level of subscales, agency was a significant positive predictor for the three positive subscales (self-kindness, common humanity, and mindfulness) and a significant negative predictor for the three negative subscales (self-judgement, isolation, and over-identification). Communion was a positive predictor only for self-kindness.

Regarding the associations between the agentic and communal facets and self-compassion, AA was a significant predictor for self-compassion on the overall level, a significant positive predictor for the three positive subscales, and a significant negative predictor for the three negative subscales. CW was a positive predictor only for self-kindness. AC and CM showed no predictive power for self-compassion at all.

## Study 2

Since the pattern of results between agency and communion with self-compassion obtained in Study 1 were partly unexpected and partly explorative, we wanted to replicate these in Study 2. Therefore, we extended our hypotheses to the facets of agency and communion and the subscales of self-compassion following the pattern of results from Study 1: We expected agency to be a significant positive predictor for the three positive subscales (self-kindness, common humanity, and mindfulness) and a significant negative predictor for the three negative subscales (self-judgement, isolation, and over-identification). We predicted communion to be a positive predictor only for self-kindness. Moreover, we hypothesized AA to be a significant predictor for self-compassion on the overall level, a significant positive predictor for the three positive subscales and a significant negative predictor for the three negative subscales. We expected CW to be a positive predictor only for self-kindness.

We additionally assessed personal and relational self-esteem. We expected AA to be the strongest predictor for personal self-esteem and that the other three facets only show additional weaker predictive power (replication of the results in the German sample of Abele et al., 2016). We hypothesized that relational self-esteem is predicted by AA, CW, and CM (replication of the results of Hauke and Abele, 2020). By additionally assessing personal and relational self-esteem we could compare the relationships between agency and communion and these three different constructs of positive self-attitude within one sample.

### Method

#### Sample

Since we wanted to replicate the pattern of results from Study 1 in an even bigger sample, we aimed for approximately 400 participants. We again distributed the invitation to the online study via emails and messaging-apps using a snowball system (different contacts than in Study 1), resulting in 443 participants. Two participants withdrew their data at the end of the study and we excluded additional seven participants because they commented at the end of the study that they had problems answering the questionnaire. This left us with a final sample of 282 women, 145 men, 2 non-binary people, and 5 people not stating their gender (*M*_age_ = 31.39 years, *SD* = 13.67, range: 18–74). Most participants were students (50.0%) or employed (37.4%). Participants were rewarded with course credit or could take part in a lottery of five monetary prizes of 10€ each. In a sample of this size, a small effect size of *f*^2^ = 0.03 can be detected.

#### Procedure

The online study took about 12 min per participant. After informed consent, participants first rated their agency and communion and then their self-compassion, personal self-esteem, and relational self-esteem. Finally, we assessed sociodemographic information.

#### Measures

Agentic and Communal Traits. Agency (*α* = 0.86, α_AA_ = 0.79, α_AC_ = 0.81) and communion (*α* = 0.85, α_CW_ = 0.81, α_CM_ = 0.74) were measured as in Study 1.

Self-Compassion. Self-compassion (*α* = 0.91) was measured as in Study 1. The reliabilities of the six subscales were as follows: self-kindness *α* = 0.83, self-judgment *α* = 0.79, common humanity *α* = 0.72, isolation *α* = 0.78, mindfulness *α* = 0.69, and over-identification *α* = 0.65.

Personal Self-Esteem. Personal self-esteem was measured with a German version (von Collani and Herzberg, 2003) of the classical Rosenberg (1965) Self-Esteem Scale consisting of 10 items (sample item “On the whole, I am satisfied with myself,” *α* = 0.90). Participants answered the items on a 5-point scale from 1 = *strongly disagree* to 5 = *strongly agree.*

Relational Self-Esteem. Relational self-esteem was measured with the scale by Du et al. (2012) consisting of 7 items (sample item: “I am a worthy member of my circle of friends,” *α* = 0.86). Participants answered the items on a 5-point Likert-scale from 1 = *strongly disagree* to 5 = *strongly agree*.

### Results

#### Preliminary analyses

The descriptive statistics and the correlations are shown in Tables 1, 2, respectively. Self-compassion was highly correlated with personal self-esteem, *r* = 0.76, *p* < 0.001, and also with relational self-esteem, *r* = 0.41, *p* < 0.001. The two components of self-esteem also showed a strong correlation to each other, *r* = 0.59, *p* < 0.001.

#### Hypothesis testing

We again calculated linear regressions with (a) agency and communion and (b) their four facets as predictors for self-compassion, its six subscales, and the two self-esteem components. The results of these 18 regressions are shown in Table 4.

**Table 4:** Self-compassion and self-esteem regressed on agency/communion and the four facets (study 2).

Predictors	Self-Compassion	Self-Kindness	Self-Judgment	Common Humanity	Isolation	Mindfulness	Over-Identification	Self-Esteem personal	Self-Esteem relational
Agency/communion									
*F*	70.22***	40.07***	38.05***	20.95***	45.93***	36.75***	33.69***	213.89***	88.41***
*R*^2^ corr.	0.24	0.15	0.15	0.08	0.17	0.14	0.13	0.50	0.29
A	0.46***	0.32***	−0.35***	0.26***	−0.38***	0.37***	−0.38***	0.65***	0.42***
C	0.09*	0.14**	−0.09	0.08	−0.08	0.03	0.03	0.13***	0.22***
Facets									
*F*	36.43***	20.96***	20.77***	10.92***	23.32***	18.49***	21.24***	108.33***	44.59***
*R*^2^ corr.	0.25	0.16	0.15	0.08	0.17	0.14	0.16	0.50	0.29
AA	0.38***	0.22***	−0.34***	0.22***	−0.30***	0.27***	−0.38***	0.47***	0.28***
AC	0.11	0.15*	−0.03	0.07	−0.12	0.13*	0.01	0.24***	0.20***
CM	0.05	−0.02	−0.05	0.01	−0.05	0.02	−0.13*	0.04	0.05
CW	0.07	0.17**	−0.07	0.10	−0.06	0.03	[0.12*]	0.12**	0.19***

Displayed are the standardized regression coefficients of linear regressions, significant β-values in brackets are suppressor effects (no significant corresponding correlations) and not to be interpreted content-relatedly.

A = agency, C = communion, AA = agency-assertiveness, AC = agency-competence, CM = communion-morality, CW = communion-warmth.

*p < 0.05; **p < 0.01; ***p < 0.001.

Supporting our hypothesis, agency and communion significantly predicted self-compassion. However, the predictive power of agency was much stronger than the one of communion. Regarding the associations between the two basic dimensions and self-compassion on the level of subscales, agency was – as predicted – a significant positive predictor for the three positive subscales and a significant negative predictor for the three negative subscales. In accordance with the hypothesis, communion was a positive predictor only for self-kindness.

Concerning the associations between the agentic and communal facets and self-compassion, AA was – as expected – a significant predictor for self-compassion on the overall level, a significant positive predictor for the three positive subscales and a significant negative predictor for the three negative subscales. Also as predicted, CW was a positive predictor only for self-kindness. Additionally, and in contrast to the results of Study 1, AC was a positive predictor for self-kindness and mindfulness and CM was a negative predictor for over-identification. The significant regression coefficient of CW for over-identification is a suppression effect (no significant corresponding correlation, see Table 2) and should therefore not be interpreted content-relatedly.

In line with our hypothesis, AA was the strongest predictor for personal self-esteem and AC and CW showed additional weaker predictive power. However, against predictions, CM was no significant predictor of personal self-esteem. As expected, relational self-esteem was predicted by AA and CW. But against predictions, CM did not predict relational self-esteem whereas AC did.

## General discussion

The present research analyzed the relationship between self-compassion and the agentic and communal self-concept. We expected self-compassion to be predicted by communion (based on theoretical considerations) as well as by agency (based on previous empirical results). In two online studies, we found very similar results: Self-compassion was strongly related to agency. This applied to all six components of self-compassion. In contrast, self-compassion was barely related to communion (no significant predictor in Study 1 and weak predictor in Study 2). Only the self-compassion component self-kindness was significantly related to communion. Regarding the facets of agency and communion, self-compassion overall and also its six components were strongly related to AA. Self-kindness was also additionally related to CW.^2^ Concerning self-esteem (Study 2), our results mirrored prior research: Personal self-esteem was dominated by agency, especially by AA (Abele et al., 2016). Relational self-esteem was predicted by agency and communion (Hauke and Abele, 2020).

### Theoretical implications

Overall, the associations between agency, communion and self-compassion match those between the basic dimensions and self-esteem, not only compared to previous research but also within the same sample (Study 2): Agency, especially AA, is crucial for a positive attitude regarding the self. The pattern of results also is consistent with studies analyzing the association between constructs akin to agency and communion which have already shown that, in individualistic countries, self-compassion is more strongly related to independence and masculinity than to interdependence and femininity (Neff et al., 2008; Patzak et al., 2017; Tatum, 2012; Yarnell et al., 2019).

A possible explanation for the strong association of agency and self-compassion could be that the self is important to individuals high in agency. Therefore, the person does their utmost to feel good which corresponds to high self-compassion. Self-compassion is not only an attitude but rather a practice toward one’s self. Because AA is the motivational facet of agency, this facet is especially important because it constitutes the drive for feeling well and taking care of one’s needs. However, these explanations are post-hoc and examining the mechanisms between the association behind agency and self-compassion is an interesting open question for further research.

The most surprising result of our study was that communion has no strong predictive value for self-compassion. This finding contradicts our theoretical considerations and also those by Neff and Vonk (2009) and Neff and McGehee (2010). While compassion (for others) is clearly related to a communal self-concept, self-compassion is not. This shows that communion might not be associated with the aspect of *caring* in general but with caring for *others*, which is in line with the definition of communion as the dimension for “getting along” (Hogan, 1982) and acting in the interest of others. In addition, our results indicate that self-compassion is not simply an intrapersonal version of the interpersonal construct of compassion (Neff and McGehee, 2010).

In a nutshell, self-compassion might be an alternative positive attitude toward the self, but no *communal* alternative to self-esteem. This conclusion is also underlined by our preliminary analyses in Study 2 showing that the correlation of self-compassion with personal self-esteem (a rather agentic construct) is even stronger than the one with relational self-esteem (a rather communal construct). Our current interpretation of this finding is that also concerning self-compassion the perspective (self vs. others) is more important than the content (compassionate, i.e., communal, practice) and for the self, agency is of utmost importance (Abele and Wojciszke, 2014). But the relationship between self-compassion and different aspects of self-esteem itself could also be an interesting field for future research.

### Practical implications

As already outlined in the theoretical introduction, self-compassion has many positive consequences for psychological functioning and health. Therefore, people strive for increasing their self-compassion. In addition to programs directly addressing self-compassion (e.g., Al-Refae et al., 2021; Arimitsu, 2016; Bluth et al., 2016; Finlay-Jones et al., 2017; Neff and Germer, 2013), the present results suggest that interventions could also aim at improving the agentic self-concept, especially AA, to increase self-compassion. For instance, interventions could teach strategies for asserting oneself in social interactions or could aim at making situations in which one was assertive more salient to the self.

### Limitations and directions for future research

First, we want to point out that some of the scales used had low reliabilities (0.60 < *α* < 0.70) so that some of the results should be interpreted with caution. Unfortunately, low reliabilities are often the case using subscales consisting only of four to five items. Regarding the global scales all reliabilities were good. Therefore, we are confident that our main results regarding the associations between agency, communion and self-compassion as well as self-esteem are robust.

A limitation of the present research concerning the association of the agentic and communal facets with self-compassion is the correlative cross-sectional nature of the studies. Thereby, causal interpretations cannot be drawn. This means, we do not know if high AA leads to high self-compassion or if high self-compassion leads to high perceptions of AA. Longitudinal as well as experimental research on the association of self-concept and self-compassion seem to be valuable next steps for future studies.

Moreover, future studies should of course investigate potential mediators and moderators of the relationship between agency/communion and a positive attitude toward oneself. Which are the mechanisms behind it? Is it goal pursuit, emotional regulation, or resilience in the eye of failures? Regarding potential moderators, research on the association between agency/communion and self-esteem could prove moderating effects of culture, religiosity, sex, and age (Gebauer et al., 2013): Agency was more important for self-esteem in agentic cultures, as well as among nonreligious individuals, men, and younger adults. In contrast, communion was more important for self-esteem in communal cultures, as well as among religious individuals, women, and older adults. Are these moderators also applicable for the association between agency/communion and self-compassion? Effects of culture could be another limitation of our research. Our two samples were both drawn in Germany, i.e., an individualistic country (Hofstede et al., 2010). In individualistic countries where people focus more on agency, the association of agency and self-compassion could be stronger than in collectivistic countries where communion is more important. Neff et al. (2008) could for example already show that while in the USA (i.e., an individualistic country) self-compassion is related to independence (akin to agency), in Thailand (i.e., a collectivistic country) self-compassion is related to interdependence (akin to communion). Therefore, analyzing the associations between agency, communion and self-compassion in a collectivistic context could reveal interesting different results. However, other research regarding the association between self-concept and self-esteem found only small cultural differences (Abele et al., 2016; Hauke and Abele, 2020): In Germany, France, Australia, Poland, China and the USA personal self-esteem was dominated by agency, especially by AA, whereas relational self-esteem was also associated with communion (in addition to agency).

## Conclusion

Our research contributes to understanding the importance of different aspects of self-concept for a positive attitude toward the self. Starting with the expectation that communion is central for self-compassion while agency is central for self-esteem, our results showed that high agency, and especially its facet assertiveness, is also crucial for high self-compassion. Thus, self-compassion seems to be an alternative, but no communal alternative to self-esteem.

## Data Availability

The datasets presented in this study can be found in online repositories. The names of the repository/repositories and accession number(s) can be found at: https://osf.io/srjf3/?view_only=db89af10ddcb45859d71b14bb514d9a9.

## Footnotes

^1^For economic reasons and because personal and relational selves are more important than the collective self (Sedikides et al., 2013; see Introduction), we focused on personal and relational self-esteem.

^2^Moreover, in Study 2, self-kindness was also related to AC, mindfulness to AC, and over-identification negatively to CM. Since these three last results were not found in Study 1, we think that they should be replicated first before interpreting them.

## References

[ref1] Abele A. E. Hauke N. (2018). “Agency and communion in self-concept and in self-esteem” in Agency and communion in social psychology. eds. Abele A. E. Wojciszke B. (London: Routledge), 52–64.

[ref2] Abele A. E. Hauke N. Peters K. Louvet E. Szymkow A. Duan Y. (2016). Facets of the fundamental content dimensions: agency with competence and assertiveness – communion with warmth and morality. Front. Psychol. 7:1810. doi: 10.3389/fpsyg.2016.01810, PMID: 27920737 PMC5118442

[ref3] Abele A. E. Wojciszke B. (2014). Communal and agentic content in social cognition: a dual perspective model. In Abele A.E. Wojciszke B. (Eds.), ADvances in experimental social psychology (50, pp. 195–255). Oxford: Academic Press

[ref4] Al-Refae M. Al-Refae A. Munroe M. Sardella N. A. Ferrari M. (2021). A self-compassion and mindfulness-based cognitive mobile intervention (serene) for depression, anxiety, and stress: promoting adaptive emotional regulation and wisdom. Front. Psychol. 12:648087. doi: 10.3389/fpsyg.2021.648087, PMID: 33828514 PMC8019776

[ref5] Arimitsu K. (2016). The effects of a program to enhance self-compassion in Japanese individuals: a randomized controlled pilot study. J. Posit. Psychol. 11, 559–571. doi: 10.1080/17439760.2016.1152593

[ref6] Bakan D. (1966). The duality of human existence. Chicago: Rand McNally.

[ref7] Barnes C. Mongrain M. (2020). A three-factor model of personality predicts changes in depression and subjective well-being following positive psychology interventions. J. Posit. Psychol. 15, 556–568. doi: 10.1080/17439760.2019.1651891, PMID: 40904582

[ref8] Baumeister R. F. Smart L. Boden J. M. (1996). Relation of threatened egotism to violence and aggression: the dark side of high self-esteem. Psychol. Rev. 103, 5–33. doi: 10.1037/0033-295X.103.1.5, PMID: 8650299

[ref9] Bem S. L. (1974). The measurement of psychological androgyny. J. Consult. Clin. Psychol. 42, 155–162. doi: 10.1037/h0036215, PMID: 4823550

[ref10] Bluth K. Blanton P. W. (2015). The influence of self-compassion on emotional well-being among early and older adolescent males and females. J. Posit. Psychol. 10, 219–230. doi: 10.1080/17439760.2014.936967, PMID: 25750655 PMC4351754

[ref11] Bluth K. Gaylord S. A. Campo R. A. Mullarkey M. C. Hobbs L. (2016). Making friends with yourself: a mixed methods pilot study of a mindful self-compassion program for adolescents. Mindfulness 7, 479–492. doi: 10.1007/s12671-015-0476-6, PMID: 27110301 PMC4838201

[ref12] Breckler S. J. Greenwald A. G. (1986). “Motivational facets of the self” in Handbook of motivation and cognition: Foundations of social behavior. eds. Sorrentino R. M. Higgins E. T. (New York: Guilford Press), 145–164.

[ref13] Cheng H. Furnham A. (2004). Perceived parental rearing style, self-esteem and self-criticism as predictors of happiness. J. Happiness Stud. 5, 1–21. doi: 10.1023/B:JOHS.0000021704.35267.05

[ref14] Crocker J. Park L. E. (2004). The costly pursuit of self-esteem. Psychol. Bull. 130, 392–414. doi: 10.1037/0033-2909.130.3.392, PMID: 15122925

[ref15] Du H. King R. B. Chi P. (2012). The development and validation of the relational self-esteem scale. Scand. J. Psychol. 53, 258–264. doi: 10.1111/j.1467-9450.2012.00946.x, PMID: 22462657

[ref16] Faul F. Erdfelder E. Lang A. Buchner A. (2007). G*power 3: a flexible statistical power analysis program for the social, behavioral, and biomedical sciences. Behav. Res. Methods 39, 175–191. doi: 10.3758/BF03193146, PMID: 17695343

[ref17] Ferrari M. Hunt C. Harrysunker A. Abbott M. J. Beath A. P. Einstein D. A. (2019). Self-compassion interventions and psychosocial outcomes: a meta-analysis of RCTs. Mindfulness 10, 1455–1473. doi: 10.1007/s12671-019-01134-6

[ref18] Finlay-Jones A. Kane R. Rees C. (2017). Self-compassion online: a pilot study of an internet-based self-compassion cultivation program for psychology trainees. J. Clin. Psychol. 73, 797–816. doi: 10.1002/jclp.22375, PMID: 27787877

[ref19] Gebauer J. E. Wagner J. Sedikides C. Neberich W. (2013). Agency-communion and self-esteem relations are moderated by culture, religiosity, age, and sex: evidence for the “self-centrality breeds self-enhancement” principle. J. Pers. 81, 261–275. doi: 10.1111/j.1467-6494.2012.00807.x, PMID: 22812669

[ref20] Hauke N. Abele A. E. (2019). Two faces of the self: actor-self perspective and observer-self perspective are differentially related to agency versus communion. Self Identity 19, 346–368. doi: 10.1080/15298868.2019.1584582

[ref21] Hauke N. Abele A. E. (2020). Communion and self-esteem: no relationship? A closer look at the association of agency and communion with different components of self-esteem. Pers. Individ. Differ. 160:109957. doi: 10.1016/j.paid.2020.109957

[ref22] Hoffman R. M. Borders L. D. (2001). Twenty-five years after the Bem sex-role inventory: a reassessment and new issues regarding classification variability. Meas. Eval. Couns. Dev. 34, 39–55. doi: 10.1080/07481756.2001.12069021

[ref23] Hofstede G. Hofstede G. J. Minkov M. (2010). Cultures and organizations: Software of the mind. New York: McGraw-Hill.

[ref24] Hogan R. (1982). “A socioanalytic theory of personality” in Nebraska symposium on motivation. ed. Page M. (Lincoln: University of Nebraska Press), 336–355. 6843718

[ref25] Hupfeld J. Ruffieux N. (2011). Validierung einer deutschen version der self-compassion scale (SCS-D) validation of a German version of the self-compassion scale (SCS-D). Z. Klin. Psychol. Psychother. 40, 115–123. doi: 10.1026/1616-3443/a000088

[ref26] Liao K. Y. H. Stead G. B. Liao C. Y. (2021). A meta-analysis of the relation between self-compassion and self-efficacy. Mindfulness 12, 1878–1891. doi: 10.1007/s12671-021-01626-4

[ref27] MacBeth A. Gumley A. (2012). Exploring compassion: a meta-analysis of the association between self-compassion and psychopathology. Clin. Psychol. Rev. 32, 545–552. doi: 10.1016/j.cpr.2012.06.003, PMID: 22796446

[ref28] Markus H. R. Kitayama S. (1991). Culture and the self: implications for cognition, emotion, and motivation. Psychol. Rev. 98, 224–253. doi: 10.1037/0033-295X.98.2.224, PMID: 40658541

[ref29] McMillan J. H. Singh J. Simonetta L. G. (1994). The tyranny of self-oriented self-esteem. Educ. Horiz. 72, 141–145.

[ref30] Neff K. D. (2003a). The development and validation of a scale to measure self-compassion. Self Identity 2, 223–250. doi: 10.1080/15298860309027

[ref31] Neff K. D. (2003b). Self-compassion: an alternative conceptualization of a healthy attitude toward oneself. Self Identity 2, 85–101. doi: 10.1080/15298860309032

[ref32] Neff K. D. (2006). The role of self-compassion in healthy relationship interactions. Paper presented at the 114th annual meeting of the American Psychological Association, New Orleans, LA.

[ref33] Neff K. D. (2011). Self-compassion, self-esteem, and well-being. Soc. Personal. Psychol. Compass 5, 1–12. doi: 10.1111/j.1751-9004.2010.00330.x

[ref34] Neff K. D. Germer C. K. (2013). A pilot study and randomized controlled trial of the mindful self-compassion program. J. Clin. Psychol. 69, 28–44. doi: 10.1002/jclp.21923, PMID: 23070875

[ref35] Neff K. D. McGehee P. (2010). Self-compassion and psychological resilience among adolescents and young adults. Self Identity 9, 225–240. doi: 10.1080/15298860902979307

[ref36] Neff K. D. Pisitsungkagarn K. Hsieh Y.-P. (2008). Self-compassion and self-construal in the United States, Thailand, and Taiwan. J. Cross-Cult. Psychol. 39, 267–285. doi: 10.1177/0022022108314544

[ref37] Neff K. D. Vonk R. (2009). Self-compassion versus global self-esteem: two different ways of relating to oneself. J. Pers. 77, 23–50. doi: 10.1111/j.1467-6494.2008.00537.x, PMID: 19076996

[ref38] Niveau N. New B. Beaudoin M. (2021). Self-esteem interventions in adults - a systematic review and meta-analysis. J. Res. Pers. 94:104131. doi: 10.1016/j.jrp.2021.104131

[ref39] Orth U. Robins R. W. Meier L. L. (2009). Disentangling the effects of low self-esteem and stressful events on depression: findings from three longitudinal studies. J. Pers. Soc. Psychol. 97, 307–321. doi: 10.1037/a0015645, PMID: 19634977

[ref40] Orth U. Robins R. W. Widaman K. F. (2012). Life-span development of self-esteem and its effects on important life outcomes. J. Pers. Soc. Psychol. 102, 1271–1288. doi: 10.1037/a0025558, PMID: 21942279

[ref41] Patzak A. Kollmayer M. Schober B. (2017). Buffering impostor feelings with kindness: the mediating role of self-compassion between gender-role orientation and the impostor phenomenon. Front. Psychol. 8:1289. doi: 10.3389/fpsyg.2017.01289, PMID: 28798714 PMC5526963

[ref42] Pepping C. A. O’Donovan A. Davis P. J. (2013). The positive effects of mindfulness on self-esteem. J. Posit. Psychol. 8, 376–386. doi: 10.1080/17439760.2013.807353

[ref43] Phillips W. J. Hine D. W. (2021). Self-compassion, physical health, and health behaviour: a meta-analysis. Health Psychol. Rev. 15, 113–139. doi: 10.1080/17437199.2019.1705872, PMID: 31842689

[ref44] Rosenberg M. (1965). Society and the adolescent self-image. Princeton: Princeton University Press.

[ref45] Schönbrodt F. D. Perugini M. (2013). At what sample size do correlations stabilize? J. Res. Pers. 47, 609–612. doi: 10.1016/j.jrp.2013.05.009

[ref46] Sedikides C. Gaertner L. Luke M. A. O'Mara E. M. Gebauer J. E. (2013). “A three-tier hierarchy of self-potency: individual self, relational self, collective self” in Advances in experimental social psychology. eds. Olson J. M. Zanna M. P. (Oxford: Elsevier Academic Press), 235–295.

[ref47] Soral W. Kofta M. (2020). Differential effects of competence and morality on self-esteem at the individual and the collective level. Soc. Psychol. 51, 183–198. doi: 10.1027/1864-9335/a000410

[ref48] Spence J. T. Helmreich R. L. Stapp J. (1974). The personal attributes questionnaire: a measure of sex role stereotypes and masculinity-femininity. JSAS Catalog Selected Documents in Psychology, vol. 4, 43.

[ref49] Suh H. Jeong J. (2021). Association of self-compassion with suicidal thoughts and behaviors and non-suicidal self injury: a meta-analysis. Front. Psychol. 12:633482. doi: 10.3389/fpsyg.2021.633482, PMID: 34122224 PMC8192964

[ref50] Tatum K. J. (2012). Adherence to gender roles as a predictor of compassion and self-com-passion in women and men. Waco, Texas: Baylor University.

[ref51] Trautwein U. Lüdtke O. Köller O. Baumert J. (2006). Self-esteem, academic self-concept, and achievement: how the learning environment moderates the dynamics of self-concept. J. Pers. Soc. Psychol. 90, 334–349. doi: 10.1037/0022-3514.90.2.334, PMID: 16536654

[ref52] von Collani G. Herzberg P. Y. (2003). Eine revidierte Fassung der deutschsprachigen Skala zum Selbstwertgefühl von Rosenberg [A revised version of the German adaptation of Rosenberg's Self-Esteem Scale.]. Z. Different. Diagn. Psychol. 24, 3–7. doi: 10.1024//0170-1789.24.1.3

[ref53] Wang T. Wu X. (2024). Self-compassion moderates the relationship between neuroticism and depression in junior high school students. Front. Psychol. 15:1327789. doi: 10.3389/fpsyg.2024.1327789, PMID: 38414883 PMC10896828

[ref54] Wojciszke B. Baryla W. Parzuchowski M. Szymkow A. Abele A. E. (2011). Self-esteem is dominated by agentic over communal information. Eur. J. Soc. Psychol. 41, 617–627. doi: 10.1002/ejsp.791

[ref55] Yarnell L. M. Neff K. D. Davidson O. A. Mullarkey M. (2019). Gender differences in self-compassion: examining the role of gender role orientation. Mindfulness 10, 1136–1152. doi: 10.1007/s12671-018-1066-1

[ref56] Ybarra O. Chan E. Park H. Burnstein E. Monin B. Stanik C. (2008). Life's recurring challenges and the fundamental dimensions: an integration and its implications for cultural differences and similarities. Eur. J. Soc. Psychol. 38, 1083–1092. doi: 10.1002/ejsp.559

[ref57] Zessin U. Dickhäuser O. Garbade S. (2015). The relationship between self-compassion and well-being: a meta-analysis. Appl. Psychol. Health Well Being 7, 340–364. doi: 10.1111/aphw.12051, PMID: 26311196

